# Impact of an intervention through Facebook to strengthen Self-esteem in nursing students*

**DOI:** 10.1590/1518-8345.3215.3237

**Published:** 2020-02-14

**Authors:** Renato Mendonça Ribeiro, João Victor Bernardi Bragiola, Letícia Palota Eid, Rita de Cássia Helú Mendonça Ribeiro, Carlos Alberto da Cruz Sequeira, Daniele Alcalá Pompeo

**Affiliations:** 1 Faculdade de Medicina de São José do Rio Preto, São José do Rio Preto, SP, Brazil.; 2 Universidade Federal de Goiás, Unidade Acadêmica Especial de Ciências da Saúde, Jataí, GO, Brazil.; 3 Escola Superior de Enfermagem do Porto, Unidade de Investigação, Porto, Portugal.

**Keywords:** Self Concept, Self Efficacy, Students, Nursing, Education, Mental Health, Nursing, Autoimagem, Autoeficácia, Estudantes de Enfermagem, Educação, Saúde Mental, Enfermagem, Autoimagen, Autoeficacia, Estudiantes de Enfermería, Educación, Salud Mental, Enfermería

## Abstract

**Objective:**

to evaluate the impact of the “Strengthening Self-Esteem” intervention proposed by the Nursing Interventions Classification, conducted through the use of Facebook, on the self-esteem and self-efficacy levels of nursing undergraduates.

**Method:**

quasi-experimental study carried out in two Higher Education Institutions. The sample consisted of 74 students. Two data collection tools were applied before and after the intervention: Rosenberg Self-Esteem Scale and General and Perceived Self-Efficacy Scale. The students were submitted to the intervention for ten sessions. Posts were made in private profile created on Facebook and consisted of positive messages, reflective texts and pictures, all supported by the persuasive resources of Bandura’s theoretical framework.

**Results:**

of the 264 students who answered the pretest, 74 (28.03%) participated in the interventions and the post-test. Rosenberg self-esteem (p=0.026) and self-efficacy (p=0.001) scores after the intervention were significantly higher than those obtained before, confirming the effectiveness of the intervention.

**Conclusion:**

the “Strengthening Self-Esteem” intervention was effective for improving students’ self-esteem and self-efficacy levels. Such interventions help spread knowledge and build mentally healthier individuals.

## Abstract (pt)

**Objetivo:**

avaliar o impacto da intervenção “Fortalecimento da Autoestima”, proposta pela *Nursing Interventions Classification*, realizada por meio do Facebook, nos níveis de autoestima e autoeficácia de graduandos de enfermagem.

**Método:**

estudo quase experimental, realizado em duas instituições de ensino superior. A amostra foi constituída por 74 estudantes. Dois instrumentos de coleta de dados foram aplicados antes e após a intervenção: Escala de Autoestima de Rosenberg e Escala de Autoeficácia Geral e Percebida. Os estudantes foram submetidos à intervenção, por dez sessões. Postagens eram feitas em perfil fechado criado no Facebook e constituíram-se por mensagens positivas, textos reflexivos e figuras, todos apoiados nos recursos persuasivos do referencial teórico de Bandura.

**Resultados:**

dos 264 estudantes que responderam aos instrumentos do pré-teste, 74 (28,03%) participaram das intervenções e do pós-teste. Os escores de autoestima de Rosenberg (p=0,026) e autoeficácia (p=0,001), após a intervenção, foram significativamente superiores aos obtidos antes, comprovando a efetividade da intervenção.

**Conclusão:**

a intervenção “Fortalecimento da Autoestima” foi efetiva para a melhora dos níveis de autoestima e autoeficácia dos estudantes. Intervenções como essas auxiliam na disseminação do conhecimento e na construção de indivíduos mentalmente mais saudáveis.

## Abstract (es)

**Objetivo:**

evaluar el impacto de la intervención “Fortalecimiento de la Autoestima”, propuesta por la *Nursing Interventions Classification*, realizada por medio del uso del Facebook, en los niveles de autoestima y autoeficacia de estudiantes del curso de enfermería.

**Método:**

estudio casi experimental, realizado en dos Instituciones de Enseñanza Superior. La muestra estuvo constituida por 74 estudiantes. Dos instrumentos de recolección de datos fueron aplicados antes y después de la intervención: Escala de Autoestima de Rosenberg y Escala de Autoeficacia General y Percibida. Los estudiantes fueron sometidos a intervención, en diez sesiones. Los *posts* de internet eran hechos en perfil cerrado creado en el Facebook y estaban constituidos por mensajes positivos, textos reflexivos y figuras, todos apoyados en los recursos persuasivos del referencial teórico de Bandura.

**Resultados:**

de los 264 estudiantes que respondieron a los instrumentos del pretest, 74 (28,03%) participaron de las intervenciones y del postest. Los puntajes de autoestima Rosenberg (p=0,026) y autoeficacia (p=0,001) después de la intervención, fueron significativamente superiores a los obtenidos antes, comprobando la efectividad de la intervención.

**Conclusión:**

la intervención “Fortalecimiento de la Autoestima” fue efectiva para la mejoría de los niveles de autoestima y autoeficacia de los estudiantes. Intervenciones como estas auxilian a diseminar el conocimiento y la construcción de individuos mentalmente más saludables.

## Introduction

Recent studies have reported that undergraduate nursing students have high stress levels^(^ 1 ^-^ 3 ^)^, low self-esteem^(^ 4 ^-^ 5 ^)^, low coping with adversities^(^ 2 ^)^ and high prevalence of suicidal ideation and behavior^(^ 6 ^)^.

This scenario warns us that these students are part of a population vulnerable to mental imbalance or crisis arising from stressors related to the undergraduate course. Physical and psychological impairments, such as anxious and depressive symptoms can occur frequently, impairing academic performance^(^ 7 ^)^, interpersonal relationships^(^ 5 ^)^, satisfaction with sleep^(^ 1 ^)^ and eating pattern^(^ 8 ^)^.

Low self-esteem is an important risk factor for several mental illnesses, especially anxiety and depression^(^ 9 ^-^ 10 ^)^. Self-esteem is the basis for an individual’s psychic construction. Liking yourself, feeling appreciation and valuing yourself is a condition associated with the development of mentally healthier young people and adults^(^ 11 ^)^.

A recent research has shown an association between self-esteem and self-efficacy^(^ 5 ^,^ 12 ^)^, identified as essential emotional support for stress relief and the adoption of satisfactory coping measures in nurses and nursing students^(^ 12 ^-^ 14 ^)^.

Self-efficacy is a key construct of Bandura’s Social Cognitive Theory and can be defined as one’s belief about one’s own competence and ability to perform and organize tasks with desired effect^(^ 15 ^)^, being associated with social skills^(^ 16 ^)^, emotional intelligence^(^ 16 ^)^ and learning processes in university students^(^ 17 ^)^.

Robust scientific evidence on interventions to promote self-esteem in university students is scarce in the literature^(^ 6 ^,^ 18 ^)^, especially with the advent of the Internet and in the Brazilian context. In general, these studies guided their interventions in group and face-to-face sessions, using many strategies such as cognitive behavioral therapy^(^ 19 ^-^ 20 ^)^, interpersonal relationship training^(^ 21 ^)^, positive self-esteem^(^ 22 ^)^ and self-esteem education assertiveness^(^ 23 ^)^.

In addition, there is little clinical research on mental health promotion using Facebook as a social network within the Internet or another technological resource. In addition, we consider this social network a favorable and prosperous strategy for positive outcomes, as studies have shown that shy people with relationship problems, lonely or with low self-esteem consider it a comfortable place to connect with others^(^ 24 ^)^.

This study addresses these gaps in the literature, proposing to test the Nursing Interventions Classification (NIC)^(^ 25 ^)^, “Strengthening Self-Esteem”, applied through the social network Facebook. The choice to work with the Internet was due to the students’ lack of time to participate in person of the activities. Among the choice of online modalities, Facebook is a popular tool widely used by young adults, has reduced cost and accessibility at any time, and produces scientific evidence, with the possibility of expansion and practical implementation.

Moreover, a randomized clinical trial that used the Internet to promote the improvement of depression pointed to the need and importance of extending investigations with online therapeutic approaches to promote psychological well-being and resilience in adults^(^ 26 ^)^.

Considering the above, the objective of our study was to evaluate the impact of the “Strengthening Self-Esteem” intervention, proposed by NIC and conducted through Facebook, on self-esteem and self-efficacy levels among nursing students.

## Method

Quasi-experimental, time-series, pre- and post-test study, conducted in two Higher Education Institutions (HEI), one public and one private, from São José do Rio Preto, São Paulo, Brazil, offering Nursing Degree.

The population consisted of all nursing students from the selected institutions (n=404). All individuals who were interested in participating were included in the research. Inclusion criteria were: being enrolled in any period of the course, being 18 or older, attending class on data collection dates, having a Facebook profile and having an electronic device to access the social network. The pre-test sample consisted of 264 students, representing 65.34% of the population.

**Figure 1:**
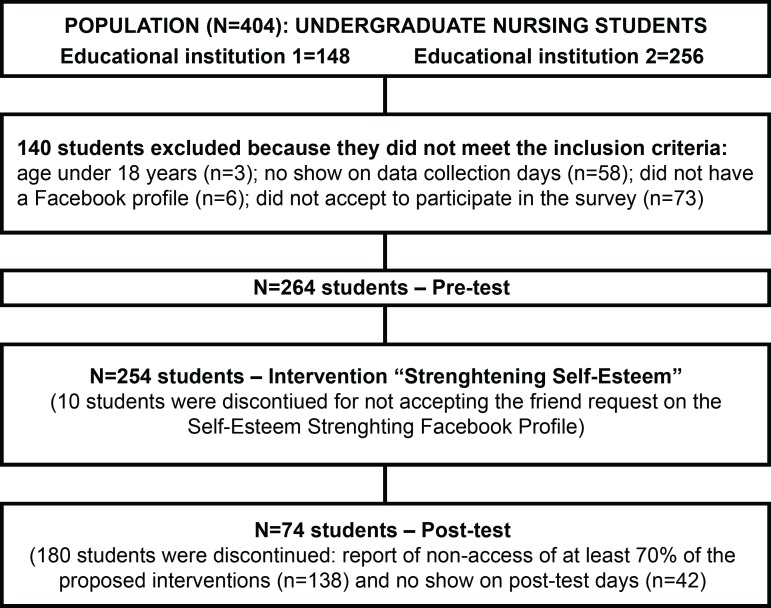
Nursing student selection and sampling process

Students who reported not having accessed at least 70% of the interventions were discontinued from the research. This value was stipulated to be able to promote changes in the individual’s beliefs, since the proposed contents were organized in a spiral, where the student saw the same topic more than once, with different ways of representation. The study was approved by the Research Ethics Committee of the São José do Rio Preto Medical School (n. 1.586.156). Informed consent was obtained from all participants. Figure 1 demonstrates the process of selection and sampling of participants.

Three data collection tools were applied: Sociodemographic Characterization, Rosenberg Self-Esteem Scale (EAR)^(^ 11 ^,^ 27 ^)^ and General and Perceived Self-Efficacy Scale^(^ 28 ^)^. The sociodemographic characterization consisted of personal, family and undergraduate course data.

EAR^(^ 11 ^)^ is a four-point Likert type; 1 means “strongly agree” and 4 “strongly disagrees”. It consists of 10 items that measure a single dimension: five that assess the individual’s positive feelings about him/herself and five negative feelings^(^ 27 ^)^. The self-esteem measure is obtained by summing the values ​​of the answers to the items and can vary from 10 to 40. The self-esteem is classified as high or satisfactory (greater than 30 points), average (from 20 to 30 points) and low or unsatisfactory (less than 20 points)^(^ 27 ^)^. This scale was validated for Portuguese in 2001^(^ 27 ^)^, has high internal consistency values ​​(Cronbach’s alpha: 0.90) and has been one of the most widely used tools in the national and international literature to assess self-esteem^(^ 29 ^)^.

The General and Perceived Self-Efficacy Scale^(^ 28 ^)^ is Likert type, consisting of 10 items, with answers ranging from one to five. These items outline the achievement of goals and the individual’s perception of a successful situation. The scale value ranges from 10 to 50. Higher score points to greater perception of self-efficacy.

The psychometric properties of the General and Perceived Self-Efficacy Scale are satisfactory in their original version (Cronbach’s alpha of 0.84) and in the validation version for Brazil (Cronbach’s alpha of 0.81), performed among university students^(^ 28 ^)^.

Data collection and intervention were carried out by researchers from February to June 2017. The intervention applied was the “Strengthening of Self-Esteem” (patient care to increase personal judgment of self-worth)^(^ 25 ^)^, using psychoeducation as a therapeutic strategy to promote higher levels of self-esteem, based on the modification of self-worth beliefs. The research nurses were the mediators of this process acting, mainly, as facilitators of knowledge construction, based on the information received. The social network Facebook was used as a resource in nurse-student interaction.

The approach to university students began in person, in the classroom, with an invitation to participate in the research. After accepting and signing the Informed Consent Form (ICF), the pretest with the three proposed data collection instruments took place. At the end of this activity, the students were informed that within a maximum of 10 days, a Facebook profile called “Strengthening Self-Esteem” would send them a friend request (based on the Facebook registration information provided by the student in the sociodemographic characterization). Consequently, individuals who still wished to participate in the intervention should accept it.

This profile “Strengthening Self-Esteem” was created as a scenario for dissemination of the proposed nursing activities. Thus, after filling out the tools, the researchers searched the student profiles on Facebook and invited them to join the intervention group.

Then, on the scheduled date, the postings about the interventions began, through the “Strengthening Self-Esteem” profile on Facebook. The content of this therapy was organized into 10 sessions, held in 10 weeks (one approach per week). The number of sessions was established from other studies^(^ 20 ^-^ 21 ^)^ and the NIC nursing activities were allocated to specific themes, as shown in Figure 2.

**Figura 2:** Sessions titles and objectives of the "Strengthening Self-Esteem" intervention

SESSION TITLE	OBJECTIVES
1. Self-knowledge and self-esteem	To encourage self-knowledge, identifying strengths and weaknesses, verbalizing positive self-affirmations, and monitoring feelings of self-negativity.
2. Overcoming self-criticism, guilt and insecurity	To assist in finding self-acceptance, avoiding criticism, and identifying the group's impact on their feelings of self-worth. To explore the reasons of self-criticism.
3. Self-confidence and self-esteem	To assist in determining your self-confidence. Reinforce positive points and avoid criticism.
4. You as protagonist of your own life	To encourage greater responsibility for yourself. To assist in assessing own behavior and monitoring the lack of follow-up in achieving goals. To encourage accepting new challenges.
5. Goal Planning	To assist in setting realistic goals. Encourage assessing own behavior, accepting new challenges, and taking greater responsibility for themselves and their goals.
6. Little challenges	To encourage acceptance of new challenges and recall successful experiences that increase autonomy.
7. See yourself in a good way	To assist in reevaluating negative self-perception and self-acceptance. To teach the strategy of rewarding and complimenting progress towards the goals achieved. To encourage identification of strengths and locus of control.
8. Do something cool	To pass on confidence in the ability to handle situations. To encourage new challenges and avoid self-criticism.
9. Avoid comparisons	To assist in overcoming bullying or teasing and assessing own behavior. To pass on confidence in the ability to handle situations.
10. Positive thinking is essential	To help identifying positive responses from others. Explore goals achieved and to pass on confidence in the ability to handle situations.

By accessing Facebook, participants would see the content of the intervention, which was preceded by a sentence that motivated them to read, such as the following example: “We have reached our 4^th^ meeting! Certainly, some changes must have already taken place within you, as you have been reading and reflecting weekly on strengthening such important social skills as self-awareness, self-esteem, self-confidence and certainty. The results will appear! This week you will be the protagonist of your life! In intervention 4, we will encourage you to accept new challenges. Challenge yourself, you are able to win. Have a great reading.” Then the students accessed the content of nursing activities.

These activities to strengthen self-esteem were offered in a text form, with positive messages, reflective texts and pictures, all supported by the persuasive resources of Bandura’s theoretical framework^(^ 15 ^)^. As a way to influence students, the material provided during all sessions contained motivational words and phrases (examples: challenges, opportunities, coping, confidence, goals, among others). The word “you” was often used as a trigger to activate persuasion because it puts the person at the core of the situation.

The content of the intervention was subjected to validation of appearance (visual, colors, letters, spaces, figures) and content (appropriateness, organization and number of sessions) by six professionals specialized in mental health and psychoeducation, as well as a newly graduated nurse from the Nursing Residency Program from one of the HEI in which data were collected, totaling seven evaluators. The small suggestions of modifications were accepted and referred to alteration in the order of sentences, substitution of words and adequacy of some objectives.

**Figure 3:**
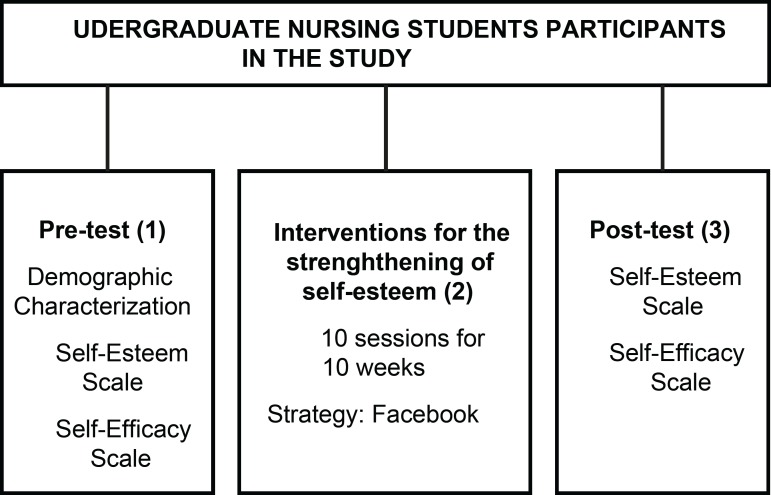
Research data collection steps

During the intervention period, we observed on Facebook several “likes” and positive comments from the university students about the content and challenges proposed in each session. There was no face interaction during the ten weeks of posts. The post-test was started one week after the conclusion of the interventions, performed in the classroom and in person. Figure 3 illustrates the data collection steps.

Data were processed and analyzed using the Minitab 17 program (Minitab Inc.). Descriptive analyzes were performed for the sample characterization variables. To analyze the influence of the sample characterization variables on the Rosenberg self-esteem scores, the t-tests for independent samples and the variance analysis test with Tukey post-hoc multiple comparison test were used.

The t-test for paired samples was used to verify the differences between Rosenberg self-esteem and self-efficacy scores in the pre- and post-intervention periods.

Data followed normality and homogeneity, verified by the Anderson-Darling and Levene test, respectively. A significance level of 5% or (p <0.05) was established.

## Results

The results showed that most undergraduate Nursing students were female (232; 87.88%), without a partner (167; 63.26%), students from a private institution (142; 53.79%), attending the second year of undergraduate school (83; 31.44%), from the municipality where the HEI is located (152; 57.58%), living in the same city as their family (192; 72.73%) and not employed (167; 63.26%).

Most students mentioned the Nursing course as the first option in the college entrance exam (n=161; 60.98%). The others (n=103; 39.02%) stated preference for other degrees: Medical school (n=61; 59.22%), followed by Law school (n=5; 4.85). Most students say they are satisfied with the course (227; 85.98%) and did not think about dropping it (n=162; 61.36%). Most reported feeling overwhelmed with undergraduate activities (n=154; 58.33%) and self-rated with good course performance (n=173; 66.03%).

Of the 264 nursing students who answered the pre-test instruments, 74 (28.03%) participated in at least 70% of the interventions and answered the post-test. The self-esteem in the pre-test was moderate (average 23.48) and was not influenced by the variables: gender, age, year of graduation, family income, satisfaction with the course, activity overload and type of institution.

Table 1 shows the comparison of the Rosenberg self-esteem and self-efficacy scores in the pre- and post-intervention periods.

**Table 1:** Descriptive statistics of Rosenberg self-esteem and self-efficacy, pre- and post- intervention of undergraduate nursing students. Sao Jose do Rio Preto, SP, Brazil, 2017

Parameter		Descriptive statistics			p value*
		N	Mean ± standard deviation	Median	
Rosenberg Self-esteem	Pre	74	23.48±2.65	2.00	0.026
	Post	74	24.32±2.19	24.00	
Self-efficacy	Pre	74	35.67±8.17	36.50	0.001
	Post	74	39.12±6.83	39.00	

* p value = for t-test for samples paired p<0.05

The results showed that all comparisons brought significant results (p<0.05). In all cases, Rosenberg self-esteem and self-efficacy, post-intervention scores were significantly higher than pre-intervention scores. This assumes that Facebook’s “Strengthening Self-Esteem” intervention had a positive effect on the self-esteem and self-efficacy levels of the students evaluated in the study.

## Discussion

Of the 264 participants in our study, 74 completed the intervention. When comparing our findings with the literature, we found that, in fact, there is greater adherence to face-to-face therapies^(^ 19 ^-^ 22 ^,^ 30 ^-^ 31 ^)^ and that most studies that tested interventions to improve mental health over the Internet presented significant losses during the process, ranging from 44.1% to 81.63%^(^ 32 ^-^ 35 ^)^.

It is considered that the low adherence to interventions may have occurred due to the constant complaints of participants’ lack of time due to academic activities and difficulties in reconciling higher education with work. Another aspect could be the unconscious distancing of the student from all situations that make him/her come into contact with their weaknesses and suffering, a condition already experienced in session 1, which addressed self-knowledge and the recognition of their strengths and weaknesses.

The results showed that the intervention tested in this research had a positive effect in strengthening the self-esteem of nursing students. The literature, although scarce on this theme, corroborates our findings^(^ 23 ^,^ 34 ^-^ 37 ^)^.

Investigations based on experimental designs^(^ 23 ^,^ 34 ^-^ 36 ^)^ that used digital technology to implement interventions and evaluate their impact on various mental conditions, such as anxiety, depression, stress, self-esteem, self-efficacy and well-being were also confirmed positive results from online interventions in improving emotional indicators.

A recent US study tested mental health promotion actions implemented through smartphone apps on 283 adults with depressive symptoms. Patients were allocated to the following groups: G1: cognitive behavioral therapy and positive psychotherapy (n=93); G2: self-esteem and self-acceptance (n=97); G3: controls (n=93) and were encouraged to access the content of the intervention for ten minutes daily for a period of one month. The results showed that depression and anxiety levels were reduced and self-efficacy levels increased in groups 1 and 2. There was no difference between groups 1 and 2, showing that intervention focused on self-esteem and acceptance was as effective as cognitive behavior therapy and positive psychotherapy^(^ 35 ^)^.

A positive psychology program, applied over the Internet for six weeks, has been tested on 235 people ages 12 to 18 to promote well-being and favorable mental health outcomes. Qualitative results pointed that 79% of participants in the experimental group had positive experiences using the website and 89% said they would continue to use the tools provided. Moreover, when compared to the controls, the study members obtained high scores for adherence to training, also recording a decrease in depression, anxiety and stress, as well as increased well-being^(^ 34 ^)^.

Similarly, an Australian study tested an online intervention for 12 weeks on 298 adults with high levels of psychological stress. After the program, quality of life, self-esteem levels, and empowerment were improved in the experimental groups. The research highlighted the benefits of applying Internet interventions to promote self-esteem and, consequently, resilience and positive coping^(^ 23 ^,^ 36 ^)^.

Reinforcing these findings, a recent meta-analysis gathered evidence that pointed to online interventions, with approaches based on cognitive behavioral therapy, as promising tools in reducing depressive and anxious symptoms in young people. In addition, it highlighted positive effects on mental health only in short-term therapies, while long-term effects characterize a gap due to the reduced number of studies^(^ 37 ^)^.

Another systematic review with meta-analysis analyzed clinical trials that tested the effect of Internet-based interventions on improving depression, anxiety and stress in college students. A total of 17 studies were found and 14 had sufficient data for meta-analysis, representing 1795 randomized participants and 1480 analyzed. The results suggested that Internet-based interventions may be effective in improving students’ depression, anxiety and stress outcomes when compared to control ones^(^ 38 ^)^.

Scientific evidence points to the effectiveness of digital media interventions, but a caution is needed, as most of these researches have been conducted with adults, many with anxiety and depression. Therefore, the results cannot be generalized to a specific population, such as our research. Thus, we also sought to compare our findings with investigations that used other resources to promote self-esteem in nursing undergraduates.

Several studies using face-to-face intervention methods that were effective in improving psychic parameters, especially self-esteem, were identified in nursing students^(^ 19 ^-^ 22 ^,^ 30 ^))^ and university students from other courses^(^ 31 ^)^. This shows that different strategies can be used to promote mental health in young people with positive results.

One of these methods was cognitive behavioral therapy, applied with the objective of promoting improvement of students’ self-esteem and self-efficacy levels^(^ 20 ^)^, stress, self-efficacy and coping strategies^(^ 19 ^)^ and self-esteem and resilience^(^ 31 ^)^. The results were effective; However, this is a specific intervention by a qualified psychologist or physician.

In the field of nursing, face-to-face strategies such as positive psychotherapy, positive self-esteem promotion and interpersonal relationships program, self-esteem and depression were tested and obtained positive results in the mental health of nursing students^(^ 21 ^-^ 22 ^,^ 30 ^)^.

The findings of this research and the set of evidence presented point to the need and importance of implementing programs for the emotional empowerment of young people by educational authorities, whether online or in person, to work on priceless values parameters such as self-esteem and self-efficacy, which can be optimized in this population.

It is also noted that most interventions to promote mental health deal with anxiety and depression^(^ 21 ^,^ 34 ^-^ 35 ^,^ 37 ^-^ 38 ^)^. This fact may be related to the high prevalence of these diseases in the world population, with the prospect of elevation^(^ 39 ^)^, a condition that encourages scientific research.

The literature has solid and consistent evidence of the association of self-esteem with emotional variables, especially depression^(^ 9 ^-^ 10 ^)^. There are several theoretical models that describe this relationship, however the most robust evidence falls on the vulnerability model, that is, low self-esteem contributes to depression^(^ 9 ^)^.

Our study did not assess anxiety and depression, however, theoretical research emphasized that self-esteem has a long-term impact on an individual’s life. In addition, the authors suggest that depression may be prevented or reduced by interventions that strengthen self-esteem^(^ 9 ^)^, data that increase the relevance of programs to strengthen this construct.

The satisfactory results achieved in this research can be explained by man’s ability to intentionally intervene in his environment. People not only react to the external environment, but have the ability to reflect on it in order to glimpse and choose action that they find most convenient or necessary^(^ 15 ^)^. These psycho-emotional interventions can offer individuals favoring environmental conditions, as well as promote personal judgment of capacity, use cognitive and metacognitive and self-reinforcing strategies^(^ 15 ^)^.

Face-to-face therapeutic proposals represent the majority of studies; however, resources from the Internet have been promising and effective opportunities for psychosocial interventions, either in the treatment and/or prevention of mental health conditions^(^ 33 ^,^ 35 ^-^ 36 ^)^. 

A study using the Web to reduce depression in adults showed that, overall, participants reported a high level of confidence in online intervention to improve a person’s understanding of depression. However, reliability that a website could help people learn skills to prevent depression was lower^(^ 36 ^)^.

A recent research on an intervention based entirely on cognitive behavioral theory called myCompass aimed at the improvement of depression, anxiety and stress revealed that most users found the interactive elements of the program useful, especially the tasks to be developed. and self-monitoring of their progress ^(^ 33 ^)^.

Facebook, in particular, is a promising tool, but little explored, for both recruiting prospective research participants and intervention methods to strengthen the mental health of its users^(^ 40 ^)^.

Compared with non-Facebook users, current results pointed that Internet users have higher values ​​of certain personality traits and positive variables that protect mental health, such as significantly higher scores on self-esteem, extroversion, social support, satisfaction with life and subjective happiness^(^ 41 ^)^. Given these findings, it can be inferred that the use of this online platform as a way of implementing interventions can enhance the positive effects of mental health.

In addition, the use of these technological resources may help the person to obtain greater involvement, engagement, responsibility and empowerment of their treatment, essential conditions for behavioral changes.

This study had limitations that should be highlighted when interpreting the results. Initially, quasi-experimental designs are not robust enough to actually establish cause and effect relationships. Knowledge about participation in an intervention may have influenced more desirable post-test responses.

Moreover, the variables measured in this study are subjective and self-reported, in which the researcher cannot confirm the reliability of the answers. Tools that measure self-esteem and self-efficacy have content directed to the way of being, acting and behaving; therefore, they require reflection and self-knowledge on the part of the respondent. In addition, despite assurance of anonymity, the participant may not be comfortable expressing feelings and beliefs of self-deprecation, devaluation, and inability to successfully perform tasks in a research.

Finally, we highlight the non-monitoring of students in periods subsequent to the post-test, so that we could verify the effect of our intervention in the medium and long term.

However, important implications for practice should be considered. This study, unpublished in Brazilian nursing students, adds a body of scientific knowledge capable of evoking discussions and reflections on the worrying panorama about the mental health of young university students and points to an accessible, easy-to-apply and evidence-based strategy to be implemented by educational authorities.

In addition, the proposed intervention, which is broad and easily accessible to young people, can be implemented and expanded to nursing courses at other universities, as well as to other university populations. From this perspective, it has the potential to be applied alone or to be associated with traditional psychotherapy, being able to reduce distances and costs to participants and educational authorities, and the extent of its impact is immeasurable.

This study revealed interesting directions for scientific research in nursing. Positive changes were elucidated in relation to the application of an intervention through social networking, in technological format, unlike traditional and presential ways. This suggests that mental health nursing interventions can be studied and optimized in similar digital formats, producing evidence-based knowledge.

Finally, a relevant contribution to the Nursing area was the use of NIC as a guide for an intervention with the Facebook device, with satisfactory results. NIC provides quality patient-centered, evidence-based care to nurses through effective, safe and efficient interventions.

## Conclusion

We conclude that the “Self-Esteem Strengthening” intervention, proposed by the NIC and applied through Facebook, was effective in raising the self-esteem and self-efficacy levels of undergraduate nursing students. Interventions such as these provide expanded access to information and can help build mentally healthier individuals.

We expect that, with the advent of new technologies, higher education schools in nursing can establish guidelines and implement actions that promote the mental health and well-being of their students.
